# Predicting the Easiness and Complexity of English Health Materials for International Tertiary Students With Linguistically Enhanced Machine Learning Algorithms: Development and Validation Study

**DOI:** 10.2196/25110

**Published:** 2021-10-26

**Authors:** Wenxiu Xie, Christine Ji, Tianyong Hao, Chi-Yin Chow

**Affiliations:** 1 Department of Computer Science City University of Hong Kong Hong Kong Hong Kong; 2 School of Languages and Cultures University of Sydney Sydney Australia; 3 School of Computer Science South China Normal University Guangzhou China

**Keywords:** feature selection, logistic regression, online health resources

## Abstract

**Background:**

There is an increasing body of research on the development of machine learning algorithms in the evaluation of online health educational resources for specific readerships. Machine learning algorithms are known for their lack of interpretability compared with statistics. Given their high predictive precision, improving the interpretability of these algorithms can help increase their applicability and replicability in health educational research and applied linguistics, as well as in the development and review of new health education resources for effective and accessible health education.

**Objective:**

Our study aimed to develop a linguistically enriched machine learning model to predict binary outcomes of online English health educational resources in terms of their easiness and complexity for international tertiary students.

**Methods:**

Logistic regression emerged as the best performing algorithm compared with support vector machine (SVM) (linear), SVM (radial basis function), random forest, and extreme gradient boosting on the transformed data set using L2 normalization. We applied recursive feature elimination with SVM to perform automatic feature selection. The automatically selected features (n=67) were then further streamlined through expert review. The finalized feature set of 22 semantic features achieved a similar area under the curve, sensitivity, specificity, and accuracy compared with the initial (n=115) and automatically selected feature sets (n=67). Logistic regression with the linguistically enhanced feature set (n=22) exhibited important stability and robustness on the training data of different sizes (20%, 40%, 60%, and 80%), and showed consistently high performance when compared with the other 4 algorithms (SVM [linear], SVM [radial basis function], random forest, and extreme gradient boosting).

**Results:**

We identified semantic features (with positive regression coefficients) contributing to the prediction of easy-to-understand online health texts and semantic features (with negative regression coefficients) contributing to the prediction of hard-to-understand health materials for readers with nonnative English backgrounds. Language complexity was explained by lexical difficulty (rarity and medical terminology), verbs typical of medical discourse, and syntactic complexity. Language easiness of online health materials was associated with features such as common speech act verbs, personal pronouns, and familiar reasoning verbs. Successive permutation of features illustrated the interaction between these features and their impact on key performance indicators of the machine learning algorithms.

**Conclusions:**

The new logistic regression model developed exhibited consistency, scalability, and, more importantly, interpretability based on existing health and linguistic research. It was found that low and high linguistic accessibilities of online health materials were explained by 2 sets of distinct semantic features. This revealed the inherent complexity of effective health communication beyond current readability analyses, which were limited to syntactic complexity and lexical difficulty.

## Introduction

For a long time, the study of the quality of language for effective health communication and education has focused on the complexity of health and medical educational resources [1-5]. A range of readability assessment tools have been developed to measure the lexical, grammatical, and syntactic features of health and educational resources [6-9]. Existing research shows that lack of linguistic understandability or readability can be explained by lexical difficulty, and complex grammatical and syntactic features [10-12]. This has caused the wide assumption that controlling for these textual features alone can help achieve the optimized reading experiences of medical and health materials for most people [13,14].

More recently, increasing research efforts have been geared toward developing accessible or easy-to-understand health materials and resources to help reduce the widening health inequality caused by socioeconomic determinants in societies with large and diverse vulnerable populations [15-19]. The key research question is whether previous studies and insights gained into health material readability can be translated directly into the design and development of accessible health resources for diverse populations, or whether there is a one-size-fits-all approach to accessible health information evaluation.

Natural language processing tools and machine learning algorithms have gained increasing popularity in health informatics. These flexible and versatile computational techniques can achieve high-precision prediction of outcomes based on the data-driven learning and computing of quantifiable features of the study object [1,7,10,20]. This represents a significant advance from statistics, which requires the presence of both dependent and independent variables to fit their relations into developed statistical models [21]. In the deployment stage, validated machine learning algorithms do not require the outcome variable to be available, as the algorithms can effectively predict the outcome, either a categorical or continuous variable, based on the computational learning of relevant features of the study object [22].

Providing reliable high-precision prediction of the outcome variable, for example, new online health information before release to the intended readers, can help identify and reduce potential barriers to health information understanding and thus increase the wide social accessibility of critical health information among diverse vulnerable populations or populations at risk due to lack of English proficiency and exposure to English health educational traditions.

Using first-hand materials from a diverse range of English health websites, our study developed a high-performing machine learning model to effectively predict the easiness versus difficulty of original English health information among young adults from nonnative English-speaking backgrounds. The machine learning algorithm revealed that while the difficulty of English health information can be explained by existing readability research, such as lexical unfamiliarity, medical terminology, and jargons, as well as syntactic complexity that can be measured by long and complex sentence structures, the easiness or understandability of original English material can be explained by distinct textual and semantic features associated with the use of common speech act verbs, familiar verbs of mental acts and processes (understand, learn, trust, feel, remember, etc), personal names and pronouns, names of social groups and communities, affiliations, people’s relations, expressions that assist with the evaluation of events, scenarios, or circumstances such as probability expressions (can, might, may be, etc), purposeful expressions that direct or draw the attention of the readers to key points of the reading material such as adverbs describing levels, and degrees.

## Methods

### Data Collection

We collected 1000 original health texts published by national and international health authorities on a wide range of health topics ranging from infectious diseases, noncommunicable diseases (like cancers, diabetes, and cardiovascular diseases), and environmental health to mental diseases, disability, and palliative care. These materials were collected and screened for their information validity. We only kept health texts published by health authorities and organizations that have extensive experience in developing and disseminating credible health information [23-25]. Private, commercial, or nonaccredited health websites were excluded manually to ensure the reliability and usability of our research findings for the development, evaluation, and prediction of public-oriented health educational resources. The collected health materials were then divided into 2 categories of easy versus difficult materials by a small group of young adults with nonnative English-speaking backgrounds. They rated the texts on a continuous scale of 0 to 10, with 0 indicating the easiest level and 10 indicating the hardest level. Their original ratings were then standardized to z scores, and the mean z score was taken as the notional value of the reading difficulty of a certain text. Lastly, we took the grand mean of the z scores of the 1000 texts and classified the entire corpus with a binary classification framework. Those below the grand mean were labeled as easy to read texts, and those above the grand mean were labeled as difficult health texts for machine learning algorithm development.

### Machine Learning Algorithm Development

#### Data Normalization

The 1000 health education articles were randomly split into training data and test data with 700 and 300 samples, respectively. The training data were used for 5-fold cross-validation to select the best hyperparameters for the machine learning algorithms, and the test data were used for evaluation and validation. The statistic distribution of the training and test data were as follows: training data, 384 difficult and 316 easy samples; test data, 162 difficult and 138 easy samples. Data normalization is necessary and essential for the machine learning algorithms to achieve good generalization and classification performance [26,27], which normalizes and scales the range of data features to prevent those features with a larger range from dominating the optimization process. It can also improve the learning process, and the range of the value of the collected health text data varies widely from a minimal value of 0 to a maximal value of 1030. Thus, we performed the following 3 normalization methods implemented in scikit-learn [28] on the data: min-max normalization, z-score normalization, and L_2_-norm normalization. Min-max normalization scales the data to a certain range like (0,1) or (−1,1). For z-score normalization, the rescaled data would have a unit variance and 0 mean. The L_2_-norm normalization scales the data samples individually to unit norm, and the sum of the squares of the data will always be up to 1. The formulas of these 3 methods are shown in equations (1), (2), and (3), respectively. For the data sample *x*, the minimum value is denoted as *x_min_* and the maximum value is denoted as *x_max_*. The mean of the data is denoted as *x_mean_*, and the SD of the data is denoted as *x_std_*.



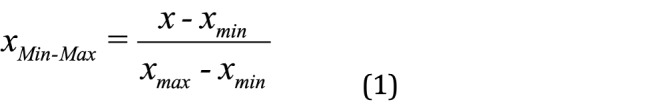





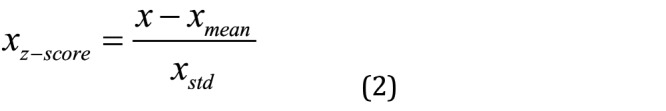





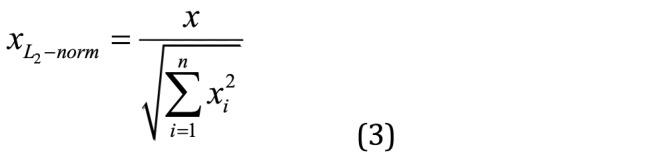



#### Hyperparameter Tuning and Model Selection

We evaluated the following 5 machine learning algorithms on our constructed health education data: linear model logistic regression (LR), linear model support vector machine (SVM) with linear kernel, nonlinear model SVM with radial basis function (RBF) kernel, ensemble tree model random forest (RF), and extreme gradient boosting (XGBoost) [29]. The nonlinear and ensemble tree models were able to learn a decision boundary that is nonlinear in the input space. The algorithms were implemented in Python with scikit-learn and xgboost packages.

**Figure 1:**
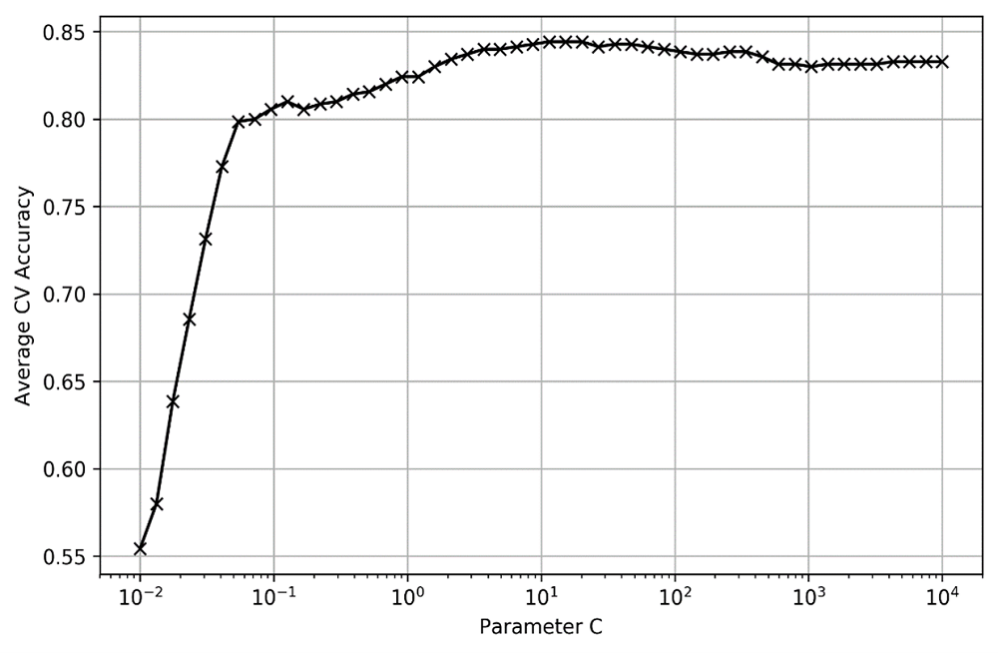
Hyperparameter tuning process of logistic regression. CV: cross-validation.

**Figure 2:**
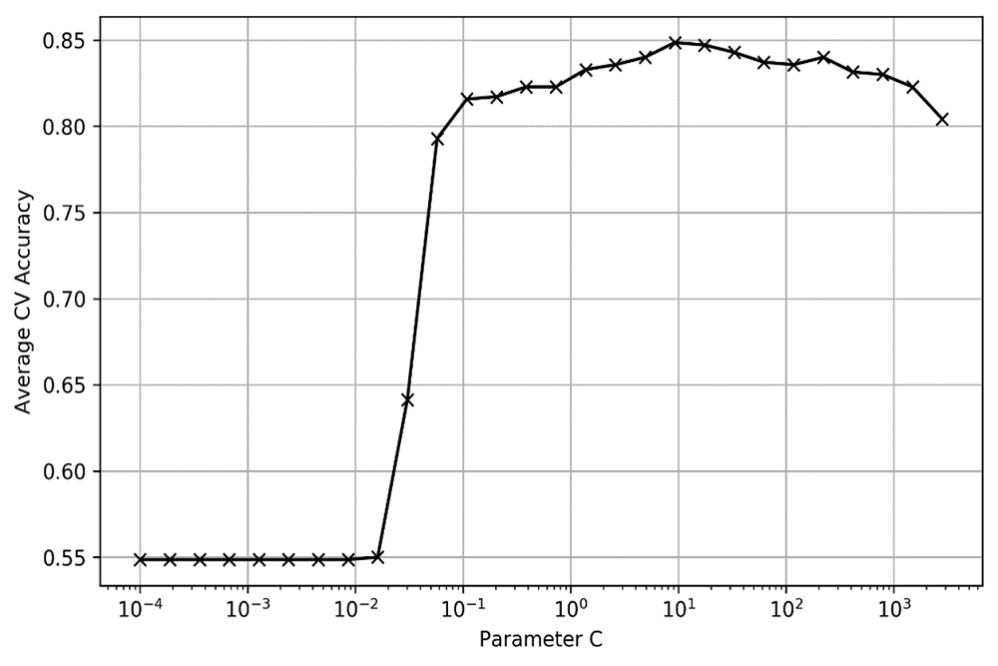
Hyperparameter tuning process of support vector machine (linear). CV: cross-validation.

To optimize the performance of the machine learning algorithms, we performed leave-one-out 5-fold cross-validation on the training data to fine tune the hyperparameters of each model via automatic grid search and randomized search methods. For LR, SVM (linear), and SVM (RBF), where the candidate values of hyperparameters are discrete, we applied a grid search to perform an exhaustive search to find the best and cross-validated parameter values of the model. For the ensemble tree model RF and XGBoost, where some of the hyperparameters are continuous, the randomized search method was applied to save the hyperparameter tuning space and time, which sampled a fixed number of parameter settings from the specified distribution instead of performing an exhaustive search. Figures 1 and 2 show the hyperparameter tuning process of the LR and SVM (linear) models, respectively. The fine-tuned values of core hyperparameters of the models are shown in Table 1. For hyperparameters not listed, we used the default value in the model.

**Table 1:** The fine-tuned values of the core hyperparameters of machine learning algorithms.

Algorithm and hyperparameter name		Description	Value
**Logistic regression**			
	C	Inverse of regularization strength	11.51395399
**Support vector machine (linear)**			
	C	Regularization parameter. The strength of the regularization is inversely proportional to C.	9.236708571873866
	kernel	The kernel type to be used in the algorithm.	linear
	probability	Whether to enable probability estimates.	True
**Support vector machine (radial basis function)**			
	C	Regularization parameter. The strength of the regularization is inversely proportional to C.	221.22162910704503
	gamma	Kernel coefficient.	0.02212216291070450
	kernel	The kernel type to be used in the algorithm.	rbf
	probability	Whether to enable probability estimates.	True
**Extreme gradient boosting (XGBoost)**			
	subsample	Subsample ratio of the training instances.	0.7842105263157895
	n_estimators	Number of boosted trees to fit.	120
	min_child_weight	Minimum sum of instance weight (hessian) needed in a child.	2
	max_depth	Maximum depth of a tree.	4
	learning_rate	Step size shrinkage used in an update to prevent overfitting.	0.11473684210526315
	colsample_bytree	The subsample ratio of columns when constructing each tree.	0.5666666666666667
**RFE_SVM^a^**			
	estimator	A supervised learning estimator with a fit method that provides information about feature importance.	SVM
	min_features_to_select	The minimum number of features to be selected.	1
	step	The number of features to remove at each iteration.	1
**Random forest**			
	n_estimators	The number of trees in the forest	100
	max_depth	The maximum depth of the tree.	4
	min_samples_leaf	The minimum number of samples required to split an internal node.	0.0463703639292683
	min_samples_split	The minimum number of samples required to be at a leaf node.	0.06216133047419098

^a^RFE_SVM: recursive feature elimination_support vector machine.

## Results

### Statistical Analyses

We annotated the corpus with the semantic annotation system developed by Lancaster University, UK [30]. The features were count data. Table 2 shows the results of the Mann-Whitney *U* test of the 2 sets of health texts across 22 of the original 115 semantic features as an illustration of contrasts between easy and difficult texts. Statistically significant differences (*P*<.05) existed for most features. To help with the understanding of the annotated semantic features, some typical words of each feature were extracted from the original corpus. A2 included cause, affect, trigger, develop, progression, depend, evoke, transmission, modify, etc. Examples from texts classified as difficult are “neurodegenerative condition that *affects* the central nervous system,” “in some cases, *evoked* potentials (nerve transmission speed) may be measured and/or a lumbar puncture (spinal tap) may be required,” and “an attack *results in* inflammation and *development* of one or more lesions, resulting in scarring (sclerotic plaque), forming on the nerves.” A7 included can, may, might, could, must, etc. Examples from texts classified as easy to understand are “the second section has an orange border and *can* help you understand what *may* have happened to you,” “you *can* ask someone you trust to help you with these books. This *might* be a disability support worker or a family violence support worker,” and “these books are about where violence *can* happen and who *can* do violence.” A12 included problems, hard, tough, etc. Examples from texts classified as easy to understand are “in fact, most kids run away due to *problems* with their families,” “anger is one of the hardest emotions to manage because it’s so strong,” “if your friend is thinking about running away, warn him or her about how tough it will be to survive on the streets.” A13 included most, at least, thoroughly, etc. Examples from texts classified as easy to understand are “thoroughly wash your hands beforehand to reduce the risk of spreading the infection to others.” A15 included risk, safe, dangerous, danger, exposure, at risk, etc. B1 included heart, muscles, chest, blood vessel, artery, ventricle, valve, cardiovascular, and contraction. B2 included arrhythmia, arteriosclerosis, abnormalities, and Down syndrome. B3 included oxygen mask, medication, antibiotics, vaccines, diagnosis, paracetamol, ibuprofen, steroids, etc. Q2 included admit, deliver, talk, speak, call, acknowledge, advice, suggest, note, question, answer, voice, etc. S2 included women, girls, children, people, workers, staff, providers, etc. S3 included partners, friends, girlfriend, boyfriend, etc. S5 included member, organization, public, community, board, group, personal, etc. T2 included begin, start to, still be, hold off on, go on and on, get going; during, all the time, end with, etc. X2 included understand, learn, trust, feel, remember, seek, check, experience, reason, inform, review, etc. X7 included want, purposes, planning, mission, aim, target, requirement, focus, etc. Y1 included radiation, x-rays, telescope, bioterrorism, tissue engineering, anatomy, laser, etc. Y2 included software, online, internet, email, computer, websites, screen, etc. Z6 included not, no, and negative. Z99 included paratyphoid, bleach based, handwashing, alcohol based, ready to eat, cross-contamination, unpasteurized, disinfect, salmonellosis, cardiologists, parainfluenza, etc.

**Table 2:** Mann-Whitney *U* test results.

Code	Definition	Easy; mean (SD)	Difficult; mean (SD)	Asymptotic significance (2-tailed) of the mean difference (*P* value)
A2	Cause and effect	9.03 (9.41)	10.64 (12.83)	.12
A7	Probability	8.55 (10.48)	4.18 (7.30)	<.001
A12	Easy/difficult	1.54 (2.35)	0.73 (1.55)	<.001
A13	Degree descriptors	4.75 (5.57)	3.96 (4.63)	.07
A15	Safety/danger	1.17 (3.37)	1.46 (3.87)	.83
B1	Anatomy and physiology	17.09 (31.84)	15.80 (21.80)	.16
B2	Health and disease	14.66 (21.20)	24.27 (33.45)	<.001
B3	Medicines and medical treatment	8.97 (14.01)	12.77 (17.93)	<.001
Q2	Speech acts	9.67 (11.76)	5.68 (8.57)	<.001
S2	People	12.17 (16.58)	9.31 (16.12)	<.001
S3	Relationship	1.72 (4.48)	0.79 (3.14)	<.001
S5	Groups and affiliation	3.05 (5.68)	1.75 (3.38)	<.001
T2	Time	2.95 (4.09)	2.41 (3.35)	.07
X2	Mental actions and processes	10.69 (11.19)	6.62 (9.35)	<.001
X7	Intention/purposes	1.78 (3.13)	3.39 (5.55)	<.001
Y1	Science and technology in general	0.19 (0.58)	0.73 (1.52)	<.001
Y2	Information technology and computing	1.33 (3.34)	0.60 (2.09)	<.001
Z1	Personal names	2.06 (4.16)	3.05 (6.90)	.51
Z5	grammatical expressions	120.84 (120.48)	136.93 (116.84)	<.001
Z6	Negative	3.01 (5.11)	4.23 (5.17)	<.001
Z8	Pronouns	54.97 (48.37)	21.14 (30.53)	<.001
Z99	Unmatched expressions	18.63 (29.12)	45.70 (54.20)	<.001

### Model Selection

After hyperparameter tuning, we compared the performance of 5 machine learning models with different normalization methods to select the best model for our further feature selection/reduction validation. We reported 5-fold cross-validation average accuracy on the training data and accuracy on the test data of all models. For 5-fold cross-validation, we applied a different random seed so the 5-fold data sets for validation would be different from the data sets for hyperparameter tuning. The results are shown in Table 3. As shown in the results, L_2_-norm normalization can improve the classification performance of the LR, SVM (linear), and SVM (RBF) models on both training data and test data. However, z-score and min-max normalization have negative impacts on model performance. For the ensemble tree model RF and XGBoost, data normalization is unnecessary in model development since the large range of feature values can help the partitioning process. LR with L_2_-norm normalization, which yielded the best performance on both training data and test data, was selected as the best model for further validation.

**Table 3:** Performance of the 5 selected models with different data normalization methods.

Classifier	Not normalized		Z-score		Min-Max		*L*_2_-norm	
	Training	Test	Training	Test	Training	Test	Training	Test
Logistic regression	0.823	0.817	0.810	0.777	0.809	0.780	0.840	0.840
Support vector machine (linear)	0.816	0.823	0.819	0.803	0.801	0.807	0.833	0.840
Support vector machine (radial basis function)	0.833	0.823	0.807	0.793	0.829	0.787	0.834	0.833
Random forest	0.796	0.803	0.796	0.803	0.796	0.803	0.789	0.830
Extreme gradient boosting	0.821	0.840	0.821	0.840	0.837	0.837	0.803	0.837

### Automatic Feature Selection: 67 Features

**Figure 3:**
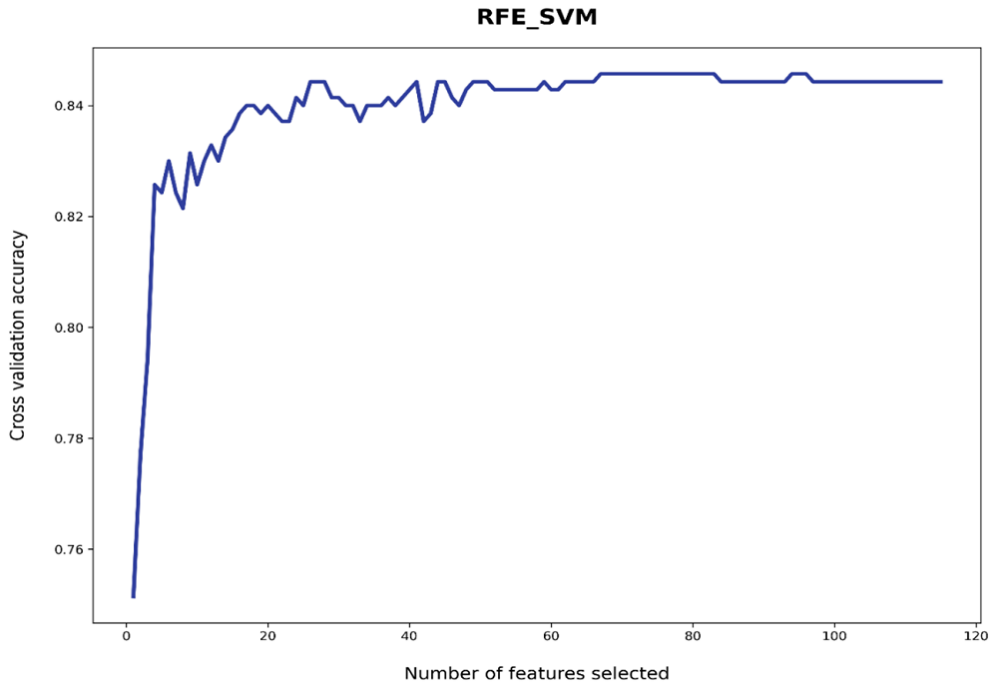
Automatic cross-validated tuning results of recursive feature elimination_support vector machine (RFE_SVM) with different numbers of selected features.

We applied recursive feature elimination (RFE) with SVM as the base estimator (RFE_SVM) to learn feature importance and performed feature reduction to remove unimportant features [31]. During the feature selection process, RFE_SVM decides whether a certain selected feature is useful or not for the SVM model to learn the decision boundary. This was achieved via iteratively eliminating features. Figure 3 shows the automatic cross-validated tuning process of RFE_SVM with different numbers of selected features. RFE_SVM learned 67 features, eliminating 48 unimportant features from the original full feature set of 115 features. The learned 67 features were automatically selected (AS) features from machine learning algorithms.

### Expert Feature Review and Refinement

Out of the 67 features selected automatically by the machine learning algorithm (RFE_SVM), 45 features were manually eliminated in the following expert review, as these features were mostly words/expressions that were not directly relevant to health or medical information. These included A1 (general actions); A5 (evaluation [good/bad, true/false]); A6 (comparison [similar/different]); A9 (getting and giving [possession]); A10 (open, finding, showing); B4 (cleaning and personal care); C1 (arts and crafts); E3 (calm/violent/angry emotions); E4 (happiness and contentment emotions); F1 (food); F2 (drinks and alcohol); F3 (smoking and nonmedical drugs); F4 (farming and horticulture); G1 (government and politics); G2 (crime, law and order); H5 (furniture and household fittings); I1 (money generally); L1 (life and living things); L2 (living creatures); L3 (plants); M1 (moving, coming, and going); M3 (vehicles, transport on land); M5 (flying/aircraft); M6 (location and direction); M8 (stationary); O2 (objects generally); O4 (physical attributes); P1 (education in general); S1 (social actions, states, processes); S7 (social actions, states, processes); S9 (religion, supernatural); T1 (time); W3 (geographical terms); and so on. The automatic feature selection reduced the original features by 41.7% from 115 to 67 features, and the subsequent expert review reduced a further 39.1% from 67 to 22 features. Tables 4 and 5 show the comparison of the performance of LR selected as the best performing algorithm with the following 3 sets of features: 115, 67, and 22. We used 70% of the data set as training data and 30% as test data, and then applied 3-fold cross-validation. The pair-wise corrected resample *t* test showed that with a significantly reduced number of features, the performance of the algorithm was not affected, even with a slightly better improvement in terms of model accuracy (mean difference of accuracy between 22 and 67 features: *P*=.04).

**Table 4:** Performance of machine learning models using different sets of features as predictors (logistic regression).

Algorithm	Accuracy, mean (SD)	Sensitivity, mean (SD)	Specificity, mean (SD)	AUC^a^, mean (SD)	Macro F1, mean (SD)
115 features	0.8400 (0.0100)	0.7767 (0.0321)	0.8933 (0.0416)	0.9188 (0.0048)	0.8367 (0.0153)
67 features	0.8400 (0.0200)	0.8333 (0.0945)	0.8333 (0.0643)	0.9177 (0.0066)	0.8367 (0.0252)
22 features	0.8567 (0.0208)	0.8100 (0.0173)	0.8933 (0.0503)	0.9108 (0.0045)	0.8567 (0.0208)

^a^AUC: area under the curve.

**Table 5:** Pair-wise corrected resampled *t* test of accuracy differences (using 3 sets of features as predictors).

Comparison			Mean difference		95% CI				*P* value (2-tailed)
					Lower		Upper		
**Pairwise comparison of accuracy**									
	Pair 1: 67 features vs 115 features	0.00%		−0.0196		0.0196		>.99	
	Pair 2: 22 features vs 115 features	1.98%		−0.0060		0.0393		.13	
	Pair 3: 22 features vs 67 features	1.98%		0.0054		0.0280		.04	
**Pairwise comparison of sensitivity**									
	Pair 1: 67 features vs 115 features	7.30%		−0.1884		0.3017		.52	
	Pair 2: 22 features vs 115 features	4.29%		−0.0265		0.0932		.20	
	Pair 3: 22 features vs 67 features	−2.80%		−0.2329		0.1862		.74	
**Pairwise comparison of specificity**									
	Pair 1: 67 features vs 115 features	−6.72%		−0.2637		0.1437		.42	
	Pair 2: 22 features vs 115 features	0.00%		−0.0392		0.0392		>.99	
	Pair 3: 22 features vs 67 features	7.20%		−0.1474		0.2674		.43	
**Pairwise comparison of AUC^a^**									
	Pair 1: 67 features vs 115 features	−0.12%		−0.0079		0.0056		.63	
	Pair 2: 22 features vs 115 features	−0.87%		−0.0264		0.0103		.28	
	Pair 3: 22 features vs 67 features	−0.75%		−0.0280		0.0142		.38	
**Pairwise comparison of F1**									
	Pair 1: 67 features vs 115 features	0.00%		−0.0196		0.0196		>.99	
	Pair 2: 22 features vs 115 features	2.39%		0.0004		0.0396		.07	
	Pair 3: 22 features vs 67 features	2.39%		0.0004		0.0396		.07	

^a^AUC: area under the curve.

### Model Validation

We evaluated the stability, robustness, scalability, and effectiveness of the 22 linguistically enhanced (LE) features. We first compared the performance of the LE features with all initial (ALL) 115 features and the 67 AS features on different sizes of training and test data. The entire data were randomly split into training data and test data with different split rates (0.2, 0.4, 0.6, and 0.8). For instance, with a split rate of 0.2 (denoted as train=0.2), 20% of data were used as training data and the remaining 80% of data were used as test data for validation. The receiver operating characteristic curve and area under the curve (AUC) metrics were used to evaluate the model performance.

**Figure 4:**
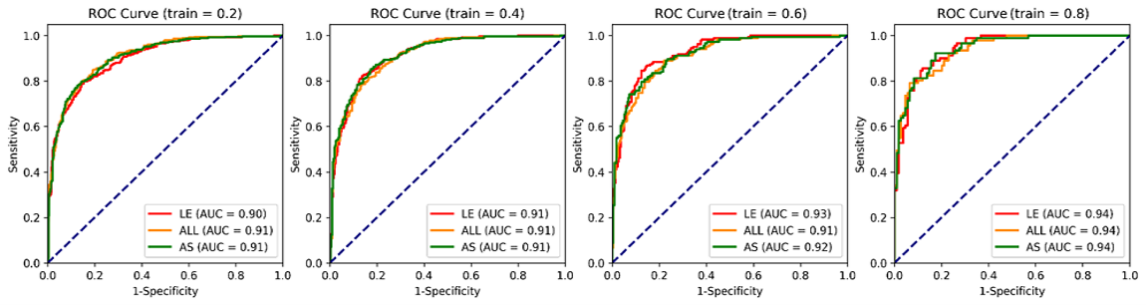
Stability and robustness of the 22 linguistically enhanced features. ALL: all initial (115 features); AS: automatically selected (67 features); AUC: area under the curve; LE: linguistically enhanced (22 features); ROC: receiver operating characteristic.

As shown in Figure 4, the model using LE features consistently yielded a comparable or higher performance on different training data set sizes (train=0.4, 0.6, and 0.8) compared with the models involving ALL and AS features. For the split rate of 0.2, the model with LE features had a lower AUC score compared with the models involving ALL and AS features. This was caused by the underfitting nature of the model involving LE features (with only 22 features, less variance, and more bias). The models involving ALL and AS features were more likely to be overfitting for the number of training data (n=200), which was very close to the number of features used for classification (115 and 67, respectively). With an increase in the training data size, the underfitting issue was solved, and the model involving LE features had better performance compared with the models involving ALL and AS features.

**Figure 5:**
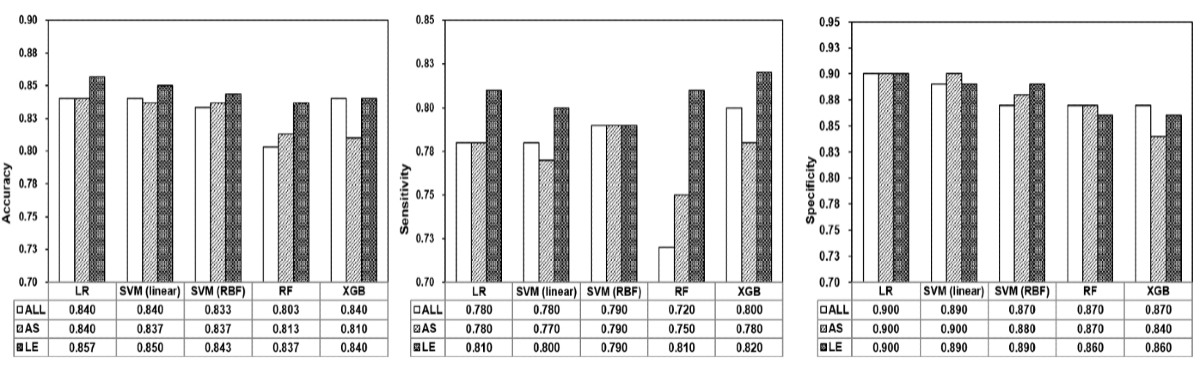
Scalability and effectiveness of the 22 linguistically enhanced features. ALL: all initial (115 features); AS: automatically selected (67 features); LE: linguistically enhanced (22 features); LR: logistic regression; RBF: radial basis function; RF: random forest; SVM: support vector machine; XGB: extreme gradient boosting.

To better evaluate the scalability, effectiveness, and contribution of the constructed LE features on health educational material classification, we compared the performance of 5 machine learning models with different feature sets. The selected machine learning models were LR, SVM (linear), SVM (RBF), RF, and XGBoost, and the models were trained on training data and evaluated on test data. The performance was assessed in terms of accuracy, sensitivity, and specificity metrics. As shown in Figure 5, LE features benefited all machine learning models compared with AS and ALL features, with a comparable or higher accuracy and sensitivity. The models (RF and XGBoost) with LE features had lower specificity than the models with AS and ALL features. Overall, LE features had a positive impact on the machine learning process, and changing the machine learning model will not affect the overall learning performance on health education data, demonstrating its scalability and effectiveness.

### Impact of Features on Model Sensitivity and Specificity

**Figure 6:**
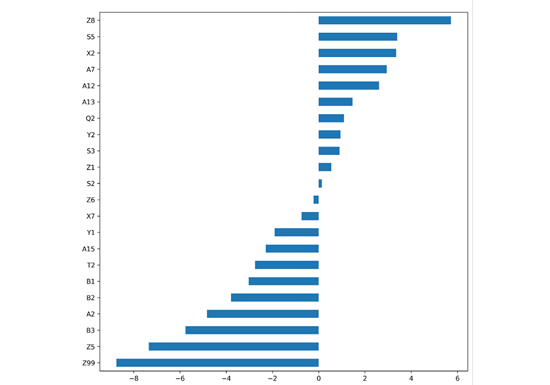
Feature coefficients in the logistic regression model with 22 semantic features. Descriptors are shown in Multimedia Appendix 1.

The final feature set contained 22 features, as presented in Figure 6, which shows the regression coefficients of the finalized 22 features. Half of the features (n=11) were associated with the easiness of the health materials, as indicated by their positive regression coefficients as follows: Z8 (pronouns), 5.717293; S5 (groups/affiliations), 3.393270; X2 (mental actions and processes), 3.350851; A7 (probability), 2.942145; A12 (easy versus difficult), 2.610647; A13 (degree descriptors), 1.462447; Q2 (speech verbs), 1.093459; Y2 (information technology/computing), 0.949234; S3 (relations), 0.898446; Z1 (personal names), 0.548855; and S2 (people), 0.135449. The other half of the features (n=11) were associated with the difficulty of the health materials, as indicated by their negative regression coefficients as follows: Z6 (negative functional words), −0.221485; X7 (intentions), −0.743811; Y1 (science and technology), −1.903669; A15 (safety/risks), −2.291032; T2 (time), −2.756571; B1 (anatomy and physiology), −3.021697; B2 (health and disease), −3.793444; A2 (cause and effect verbs), −4.838672; B3 (medicines and medical treatment), −5.763809; Z5 (grammatical expressions), −7.348969; and Z99 (unmatched or out-of-dictionary expressions), −8.749430.

**Figure 7:**
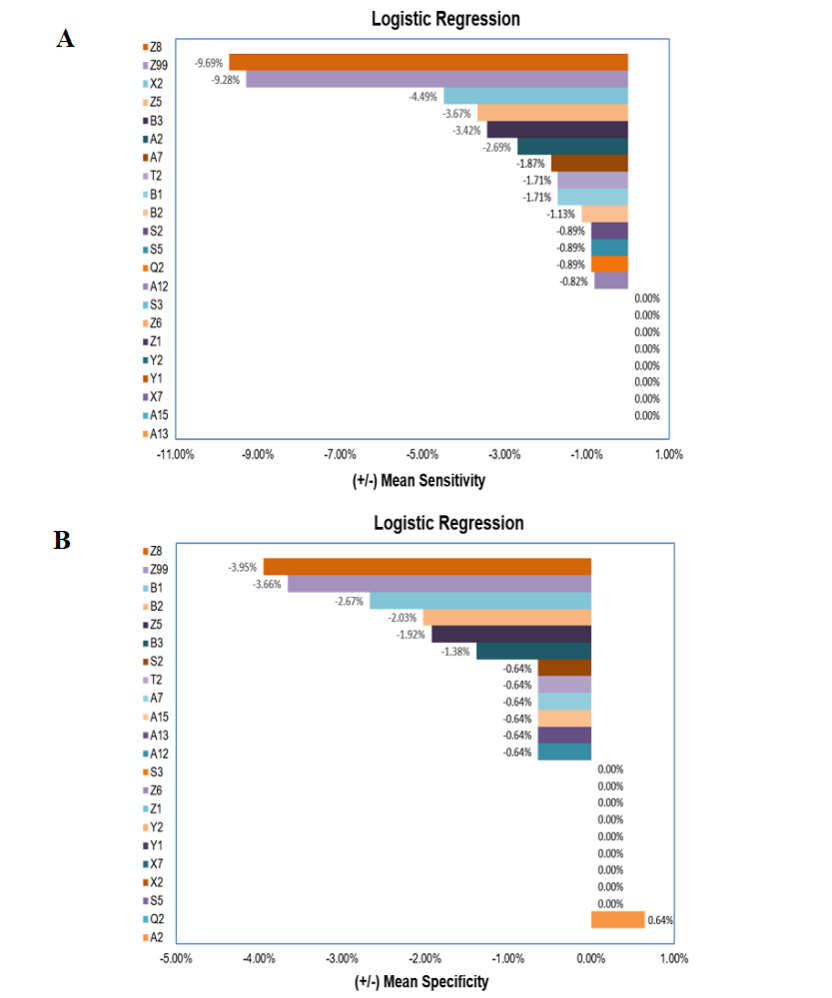
Impact of the features on model sensitivity (A) and specificity (B). Descriptors are shown in Multimedia Appendix 1.

Figure 7A shows the impact of individual features on the sensitivity of the LR algorithm. The removal of Z8 (pronouns) resulted in a mean decrease in sensitivity of 9.69% (*P*=.047; 95% CI of mean difference −0.1387 to −0.0189). The removal of A2 (cause and effect) reduced the model sensitivity by 2.69% to a mean sensitivity score of 0.7907 (SD 0.0163; *P*=.003; 95% CI −0.0256 to −0.0181). Another feature that caused a statistically significant drop in model sensitivity was X2 (mental actions and processes). The deletion of this feature resulted in a mean sensitivity decrease of 4.49%, from a mean sensitivity score of 0.8126 to 0.7761 (SD 0.014; *P*=.04; 95% CI −0.0625 to −0.0104). The following 11 features also caused decreases in the model mean sensitivity, but the changes were not statistically significant: A12 (easy/difficult; mean difference −0.82%, change to 0.8059; *P*=.42; 95% CI −0.0293 to 0.016); A7 (probability; mean difference −1.87%, change to 0.7974; *P*=.18; 95% CI −0.041 to 0.0107); B1 (anatomy and physiology; mean difference −1.71%, change to 0.7986; *P*=.18; 95% CI −0.0376 to 0.0098); B2 (health and diseases; mean difference −1.13%, change to 0.8034; *P*=.69; 95% CI −0.0773 to 0.0589); B3 (medicines and medical treatment; mean difference −3.42%, change to 0.7847; *P*=.18; 95% CI −0.0752 to 0.0195); Q2 (speech act verbs; mean difference −0.89%, change to 0.8053; *P*=.42; 95% CI −0.0318 to 0.0174); S5 (groups and affiliations; mean difference −0.89%, change to 0.8053; *P*=.42; 95% CI −0.0318 to 0.0174); Z5 (grammatical expressions; mean difference −3.67%, change to 0.7828; *P*=.08; 95% CI −0.0601 to 0.0005); Z99 (unmatched/out-of-dictionary words; mean difference −9.28%, change to 0.7371; *P*=.16; 95% CI −0.1899 to 0.039); T2 (time; mean difference −1.71%, change to 0.7986; *P*=.18; 95% CI −0.0376 to 0.0098); and S2 (people; mean difference −0.89%, change to 0.8053; *P*=.42; 95% CI −0.0318 to 0.0174). Figure 7B shows the impact of features on the specificity of the LR model. Decreases in specificity with removal were noted for the following features: A12 (easy/difficult; mean difference −0.64%, change to 0.8907; *P*=.42; 95% CI −0.0253 to 0.0138); A13 (degree descriptors; mean difference −0.64%, change to 0.8907; *P*=.42; 95% CI −0.0253 to 0.0138); A15 (safety/risks; mean difference −0.64%, change to 0.8907; *P*=.42; 95% CI −0.0253 to 0.0138); A7 (probability; mean difference −0.64%, change to 0.8907; *P*=.42; 95% CI −0.0253 to 0.0138); B1 (anatomy and physiology; mean difference −2.67%, change to 0.8725; *P*=.25; 95% CI −0.075 to 0.0272); B2 (health and diseases; mean difference −2.03%, change to 0.8783; *P*=.21; 95% CI −0.0521 to 0.0158); B3 (medicine and medical treatments; mean difference −1.38%, change to 0.884, *P*=.19; 95% CI −0.0337 to 0.0088); Z5 (grammatical expressions; mean difference −1.92%, change to 0.8792; *P*=.42; 95% CI −0.0758 to 0.0413); Z8 (pronouns; mean difference −3.95%, change to 0.861; *P*=.31; 95% CI −0.1238 to 0.053); Z99 (unmatched expressions; mean difference −3.66%, change to 0.8637; *P*=.32; 95% CI −0.1178 to 0.0522); T2 (time; mean difference −0.64%, change to 0.8907; *P*=.42; 95% CI −0.0253 to 0.0138); and S2 (people; mean difference −0.64%, change to 0.8907; *P*=.42; 95% CI −0.0253 to 0.0138). The only feature that caused an increase in specificity with its removal was A2 (cause and effect; mean difference 0.64%, change to 0.9022; *P*=.42; 95% CI −0.0138 to 0.0253).

## Discussion

### Principal Findings

Improving the readability and accessibility of online English health education resources can have important impacts on the development of health literacy and the self-health management skills of readers. Young adults represent a large and increasing group of online health information consumers. Our study developed machine learning algorithms to predict the linguistic easiness versus difficulty for international tertiary students with non-English speaking backgrounds. We first compared and selected algorithms through data normalization (L_2_-norm). LR emerged as the best performing algorithm compared with SVM, RF, and XGBoost when trained on normalized data. We used RFE with SVM as the base estimator to automatically reduce the high-dimensional feature space. The automatic feature selection reduced the original feature set (n=115) by around 40% to 67.

The subsequent expert evaluation resulted in another 40% reduction in features. The distribution of regression coefficients aligns well with the statistical analyses. The following features with positive regression coefficients in machine learning had significantly higher means in easy-to-understand health materials (Mann-Whitney *U* test): Z8 (*P*<.001), S5 (*P<*.001), X2 (*P*<.001), A7 (*P*<.001), A12 (*P*<.001), Q2 (*P*<.001), Y2 (*P*<.001), S3 (*P*<.001), and S2 (*P*<.001). only the following 2 semantic features had statistically similar means in easy and difficult texts, with positive regression coefficients: A13 (*P*=.07) and Z1 (*P*=.51). The following features with negative regression coefficients had statistically higher means in difficult health materials: Z99 (*P*<.001), Z5 (*P*<.001), B3 (*P*<.001), B2 (*P*<.001), Y1 (*P*<.001), X7 (*P*<.001), and Z6 (*P*<.001). The following 4 semantic features had a statistically similar distribution in easy and difficult texts, with negative regression coefficients: A2 (*P*=.12), A15 (*P*=.83), B1 (*P*=.16), and T2 (*P*=.07). This suggests that statistical significance is not the only determinant in the development of LR algorithms. Feature interaction may also impact the performance of algorithms, although the impact of individual features on model sensitivity and specificity was not statistically significant.

To assess the impact of features on model performance, we conducted successive permutation of features to examine changes in the sensitivity and specificity of the LR algorithm. The LR model with the 22 optimized features achieved the highest sensitivity (mean 0.813, SD 0.018) and the highest specificity (mean 0.896, SD 0.050), when compared with other feature sets, which were optimized either automatically or statistically. Within the best performing model, the following 3 semantic features caused a statistically significant decrease in model sensitivity for predicting the linguistic easiness of online health information: Z8 (pronouns), A2 (words describing causes, effects, or causal relations), and X2 (words describing mental status, actions, or processes). We interpreted this important finding in light of the impact of an information logic sequence on reading experiences. The use of pronouns and words describing the causal relations can significantly increase the explicitness of the logical structure of health information [32-34]. The addition of words describing mental status, actions, and processes can help with the reasoning and mental processing of health information [35-38]. Different from the impact on sensitivity, none of the individual features caused a statistically significant decrease in specificity for predicting the difficulty of health materials for international tertiary students. This finding correlates well with existing research on readability. Linguistic features, such as word length, word frequency, and word familiarity, and other structural features have proven to be highly relevant and reliable predictors of textual complexity and difficulty [39].

### Limitations

Our study developed an LR algorithm with a small number of features to predict the easiness and difficulty of online health information. The intended users were young adults with university degrees but with nonnative English-speaking backgrounds. The model is limited to this user group. The extensibility of our study findings to other user groups and online health materials in other languages remains to be tested and validated. Another limitation of our study is that the LR model using the finalized 22 features did not achieve statistically significant improvement over the LR model using the 115 and 67 features identified automatically. In future research, we will explore models that can achieve better performance with a small set of features that are linguistically meaningful and significant as well.

### Conclusion

We developed a high-performing LR algorithm with a small number of semantic features to predict the easiness versus difficulty of online English health resources for young adults (tertiary students) with nonnative English-speaking backgrounds. We found that reducing the number of features is essential to prevent overfitting, since models with less features are less likely to have overfitting issues. Furthermore, machine learning models with less features are less complex, are more interpretable, and have better generalization [40]. The result also demonstrates the stability and robustness of the algorithm with linguistically relevant features. Our study shows that incorporating linguistic knowledge and machine learning–aided feature selection to reduce the feature space can help develop more efficient and less complex models with a good generalization ability.

## Appendix

Multimedia Appendix 1 Notations and definitions of semantic features.

## Abbreviations

ALL: all initial
AS: automatically selected
AUC: area under the curve
LE: linguistically enhanced
LR: logistic regression
RBF: radial basis function
RF: random forest
RFE: recursive feature elimination
SVM: support vector machine
XGBoost: extreme gradient boosting
