# Supplementary material for: Predicting the Easiness and Complexity of English Health Materials for International Tertiary Students With Linguistically Enhanced Machine Learning Algorithms: Development and Validation Study
Source: JMIR Med Inform. 2021 Oct 26;9(10):e25110. doi: 10.2196/25110 (PMC8579219; doi:10.2196/25110)
Supplement: Multimedia Appendix 1 [file medinform_v9i10e25110_app1.docx]

**Multimedia Appendix** **1**. Notations and definitions of semantic features.

| **Notation** | **Definition** | **Notation** | **Definition** |
| --- | --- | --- | --- |
| **A12** | easy versus difficult | **S5** | groups and affiliations |
| **A13** | degree descriptors | **T2** | time |
| **A15** | safety or risks | **X2** | mental actions and processes |
| **A2** | cause and effect verbs | **X7** | intentions and purposes |
| **A7** | probability | **Y1** | science and technology |
| **B1** | Anatomy and physiology | **Y2** | information technology and computing |
| **B2** | health and disease | **Z1** | personal names |
| **B3** | medicines and medical treatment | **Z5** | grammatical expressions |
| **Q2** | speech act verbs | **Z6** | negative functional words |
| **S2** | people | **Z8** | pronouns |
| **S3** | relations | **Z99** | unmatched or out-of-dictionary expressions |
